# Population-Based Monitoring of Emerging HIV-1 Drug Resistance on Antiretroviral Therapy and Associated Factors in a Sentinel Site in Cameroon: Low Levels of Resistance but Poor Programmatic Performance

**DOI:** 10.1371/journal.pone.0072680

**Published:** 2013-08-26

**Authors:** Serge C. Billong, Joseph Fokam, Avelin F. Aghokeng, Pascal Milenge, Etienne Kembou, Ibile Abessouguie, Flore Beatrice Meva’a-Onglene, Anne C. Zoung-Kanyi. Bissek, Vittorio Colizzi, Eitel N. Mpoudi, Jean-Bosco N. Elat, Koulla S. Shiro

**Affiliations:** 1 National HIV Drug Resistance Prevention and Surveillance Working Group, Yaounde, Cameroon; 2 National AIDS Control Committee, Central Technical Group, Yaounde, Cameroon; 3 Faculty of Medicine and Biomedical Sciences of the University of Yaounde 1, Yaounde, Cameroon; 4 CIRCB: Chantal BIYA International Reference Centre for Research on HIV/AIDS prevention and management, Molecular Biology Laboratory, Sequencing unit, Yaounde, Cameroon; 5 Centre de Recherche en Maladies Emergentes et Ré-émergentes (CREMER)/IMPM/IRD, Virology Laboratory, Yaounde, Cameroon; 6 Institut de Recherche pour le Développement (IRD), University Montpellier 1, UMI 233 TransVIHMI, Montpellier, France; 7 World Health Organisation (WHO), Afro, HIV/AIDS, Yaounde, Cameroon; 8 National Social Insurance Centre Hospital, Yaoundé-Cameroon, Approved Treatment Centre (ATC), Yaounde, Cameroon; 9 Department of Disease Control, Ministry of Public Health, Yaounde, Cameroon; 10 University of Rome Tor Vergata, Laboratory of Immunology and Molecular Pathology, Department of Biology, Faculty of Sciences, Rome, Italy; 11 Yaounde Central Hospital, Infectiology service, Yaounde, Cameroon; 12 Division of Operational Health Research, Ministry of Public Health, Yaounde, Cameroon; 13 General Secretariat, Ministry of Public Health, Yaounde, Cameroon; 14 Agence Nationale pour la Recherche sur le Sida et les hépatites virales (ANRS)-Sud, Yaounde, Cameroon; Alberta Provincial Laboratory for Public Health/University of Alberta, Canada

## Abstract

**Background:**

Scale-up of antiretroviral therapy (ART) in resource-limited settings has drastically reduced HIV-related morbidity and mortality. However, challenges in long-term ART, adherence and HIV drug resistance (HIVDR) itself, require monitoring to limit HIVDR emergence among ART-experienced populations, in order to ensure regimen efficacy.

**Methods:**

A longitudinal study was conducted from 2009–2011 in a cohort of 141 HIV-infected adult patients (aged >21) at the national social insurance centre hospital in Yaounde, Cameroon. As per-WHO HIVDR protocol, HIV-1 protease-reverse transcriptase genotyping was performed at baseline and at endpoint (12 months) on first-line ART using ViroSeq™ Genotyping kit.

**Results:**

At baseline, a prevalence of 3.6% (5/139) HIVDR was observed [protease inhibitors M46I (1/5), G73A (1/5), L90LM (1/5); nucleoside reverse transcriptase inhibitors: M184V (1/5), T215F (1/5); non-nucleoside reverse transcriptase inhibitors: K103N (1/5), Y181Y/C (2/5), M230ML (1/5)]. At endpoint, 54.0% (76) patients were followed-up, 9.2% (13) died, and 3.5% (5) transferred, 38.5% (47) lost to follow-up (LTFU). 69.7% (53/76) of those followed-up had viremia <40 copies/ml and 90.8% (69/76) <1000 copies/ml. 4/7 patients with viremia ≥1000 copies/ml harbored HIVDR (prevalence: 5.3%; 4/76), with M184V/I (4/4) and K103K/N (3/4) being the most prevalent mutations. LTFU was favored by costs for consultation/laboratory tests, drug shortages, workload (physician/patient ratio: 1/180) and community disengagement.

**Conclusions:**

Low levels of HIVDR at baseline and at endpoint suggest a probable effectiveness of ART regimens used in Cameroon. However the possible high rate of HIVDR among LTFUs limited the strengths of our findings. Evaluating HIVDR among LTFU, improving adherence, task shifting, subsidizing/harmonizing costs for routine follow-up, are urgent measures to ensure an improved success of the country ART performance.

## Introduction

The global AIDS control strategy has made great progress in responding to the epidemics, with more people than ever receiving antiretroviral therapy (ART), care and support. The prevention revolution is delivering dramatic results while science is offering new hope [1]. Of note, a decade of ART has transformed HIV-infection from a death sentence to a manageable chronic disease [2].

In the entire low- and middle-income countries, more than 8 million people living with HIV were receiving ART in 2011, up from 6.6 million people in 2010, representing an increase of more than 20%, and up to 54% [50–60%] coverage of eligible patients based on World Health Organisation (WHO) guidelines (CD4≤350 cells/µl). Thus, there is real hope to eliminate new infections and to achieve the target of “*15 million people on ART by 2015*” [3]. In Sub-Saharan Africa, HIV prevention, treatment, care and support services have been rapidly scaled up in the WHO African Region, with 6.2 million people receiving ART in this Region by the end of 2011, representing about 40% ART coverage among eligible individuals and above half of HIV-infected pregnant women receiving ART for preventing mother-to-child transmission [4], [5]. Consequently, the efficiency and effectiveness of AIDS treatment programs need to be sustainable by setting-up essential models, not only to enhance access to ART, but also to monitor and limit the emergence and spread of HIV drug resistance (HIVDR) among individuals receiving first line drugs, which still account for the greatest majority of antiretrovirals commonly used for therapeutic management in resource-limited settings (RLS) [6].

In Cameroon, scale-up of ART started since 2003 through efforts led by the “*WHO 3 by 5*” initiative and the “*Universal access to comprehensive treatment, care and support”* launched in 2005 by the global fund to fight against AIDS, tuberculosis and malaria [7], [8]. Management of people living with HIV in this sub-Saharan African country is based on public health approaches recommended by the WHO for adult/adolescent standardized first- (two nucleoside reverse transcriptase inhibitors [NRTI], plus one non-nucleoside reverse transcriptase inhibitor [NNRTI]) and second-line (one ritonavir boosted protease inhibitor [PI/r], plus two NRTIs) regimens, which account for drug regimens currently available and used nationwide [9], [10]. Despite the increasing rate of HIVDR in both drug-naïve and ART-experienced patients [11]–[13], patients are mostly monitored based on clinical parameters, due to the limited accessibility to biological monitoring. Indeed, flow cytometry for CD4 cells count is available but still far from attaining the target, while viral load testing is less available (cost being entirely at the patient’s responsibility). Most importantly, very few laboratories are known to offer a platform for HIV-1 genotypic resistance testing (GRT), at a lower but still unaffordable cost to the greatest majority of patients (cost being entirely at the patient’s responsibility) [14], [15]. Despite a decreasing national HIV prevalence (from 5.5% in 2004, to 4.3% in 2011), the country still experienced a generalized HIV epidemiology, with ∼50% (117,000) eligible patients receiving ART [16], [17]. Since scale-up of ART is associated with high risks of HIVDR [11], strategies to minimize HIVDR are of major public health priority in Cameroon [18]–[20]. It therefore appeared essential to evaluate the extent at which HIVDR could affect the effectiveness of ARV drugs among patients newly enrolled on first line drug regimens, and to identify ART programmatic factors and service deliveries that could contribute to the emergence of HIVDR among Cameroonian patients receiving treatment [21]. Such survey may be of paramount importance to support ongoing efforts in ART scale-up led by the national AIDS control strategic plan for a sustainable, scalable, and successful ART programme performance [21]. Moreover, as significant increases in HIVDR have been reported among ART-naive patients in other African regions (29% per year in East-Africa, 14% per year in Southern Africa), against lower rate (3% per year) in west- and central-Africa [22], it would be vital for Cameroon, as any other west- and central-African country, to contribute in maintaining its current low regional rate of HIVDR through a regular national surveillance system, an important component both for the regional and global HIVDR prevention, monitoring and surveillance strategies [23].

Specifically, our current study aimed at: (1) evaluating the rate of HIVDR at baseline (prior to patient enrollment on ART); (2) estimating the proportion of patients presenting virologic success, defined as a plasma viral load <1000 copies/ml after 12 months of ART initiation (expected rate: ≥70%); (3) identifying programmatic factors potentially contributing to virologic success or failure; (4) identifying HIVDR mutations associated with virologic failure; (5) evaluating the impact of identified mutations on second and third lines ART regimens; (6) evaluating adequacy to the WHO-recommendations for standardized treatment guidelines and the simplified monitoring approach of patients receiving ART within the national context.

## Methods

### Study Design and Population

A prospective and longitudinal survey was conducted from 2009 through 2011 in a cohort of 141 HIV-infected individuals at the approved treatment centre (HIV clinic) of the national social insurance centre hospital in Yaounde, Cameroon. As per-WHO recommendations for HIVDR monitoring survey at a sentinel site, this HIV clinic was selected as the study sentinel site based on its long-term experience on patients management with ART, its conformity to the national ART guidelines, and its ability to enroll at least 130 patients on ART in a three-months period (i.e. required WHO target sample size for this study). The study participants were consecutively enrolled at their follow-up visit based on inclusion criteria (aged >15 years old; eligible for enrollment on first line ART at the studied HIV clinic; not previously enrolled on ART, and not being transferred from another HIV clinic). This cohort was monitored for 12 months following ART initiation, with an additional period of three months to monitor cases of lost to follow-up (LFTU). A standardized questionnaire was administered to assess demographic, epidemiologic, clinical, treatment, and/or adherence information at month zero (M_0_) and month twelve (M_12_) after ART initiation.

At baseline [enrollment on ART (M_0_)], HIV-1 protease-reverse transcriptase GRT was performed; while at M12 both HIV-1 plasma viral load measurements and HIV-1 protease-reverse transcriptase GRT (if plasma viral load≥1000 copies/ml) were performed for attendees.

### Sample Processing

Following consent and questionnaire administration, samples were processed at M_0_ (for GRT) and at M_12_ (both for plasma viral load and eventually for GRT) at the virology laboratory of CREMER/IMPM/IRD in Yaounde, Cameroon. A total of 10 mL whole blood was collected from each study participant in EDTA tubes, followed by centrifugation; and plasma aliquots were frozen at 80°C until use. Viremia was performed using the Abbott Applied Biosystem platform (*Real Time PCR AB m2000RT*) as-per the manufacturer instructions. HIV-1 GRT was performed using an FDA-approved commercial genotyping kit (ViroSeq™ genotyping system; Abbott Laboratories). Briefly, RNA was extracted using a commercial kit (QIAmp Viral RNA mini-kit, Qiagen Inc., USA), retrotranscribed by murine leukemia virus RT, and amplified with Amplitaq-Gold polymerase enzyme by using two different sequence-specific primers for 40 cycles. RT-PCR was regularly launched with a positive and a negative PCR control. *Pol*-amplified products (containing the entire 99 amino acids of the protease and the first 330–335 amino acids of the reverse transcriptase open reading frame, for ∼1,300 nucleotides) were directionally full-length sequenced (i.e. in sense and antisense) on an automated sequencer (ABI 3130 Applied Biosystems, Foster City, CA), using seven different overlapping sequence-specific primers. Sequences analysis was performed using SeqScape-v.2.5 software, and complete sequences encompassing the *pol* region of interest (∼1.3 kb) were assembled and manually edited. Sequences having a mixture of wild type and mutant residue(s) at single positions were considered to have the mutant(s) at the respective position. Generated sequences were submitted to Genbank under the following accession numbers (KF192112–KF192257).

### Analysis and Interpretation of HIV Drug Resistance Mutations (DRMs)

As recommended by the WHO protocols for the study of transmitted and acquired HIVDR, sequences generated at M_0_ were analyzed for HIVDR-associated mutations using the WHO updated list for the “*Surveillance of Transmitted HIV-1 Drug-Resistance Mutations*” [24]; while at M_12_ of ART, GRT was performed among patients experiencing virologic failure (plasma viral load ≥1000 copies RNA/ml) and drug resistance mutations (DRMs) were defined following the algorithm of the French HIV drug resistance database, ANRS.05.2011, (http://www.hivfrenchresistance.org/).

### Statistical Analysis

Descriptive statistics were performed for socio-demographic, clinical, immunologic and virologic data. Median and interquartile ranges (IQR) for non-normally distributed variables were reported. The Mann Whitney test was used to compare quantitative variables, with a value of p≤0.05 considered statistically significant. All the analyses were processed using SPSS statistical software, and categorical data were analyzed using the Fisher exact test, with a false discovery rate of 0.05 used to determine statistical significance.

### Ethical Considerations

Ethical clearance was obtained from the National Ethics Committee (CNE/2008) of Cameroon. At the ART site (HIV clinic), an information notice was administered to each eligible patient, who then provided an informed consent prior to enrollment into the study. This informed consent was verbal, rather than written, because patients were monitored as per the national ART programme (i.e. during their normal consultation appointments). Moreover, since our aim was to evaluate acquired HIVDR and associated programmatic factors, verbal consent was the most applicable approach in minimizing a change in patient’s routine behavior/adherence throughout the study, and in identifying the routine functionality (i.e. strengths and weaknesses) of the ART programme at the HIV clinic. To this effect, informed consent was documented in the patient’s medical files throughout the study; a procedure approved by the clearance. Confidentiality was secured by the use of unique identifiers allocated to each of the study participants. Results were beneficial both for individualized patient management and for the ART performance evaluation.

Because all our participating patients were >21 years old (i.e. adults) and without any other vulnerability, informed consent from the next of kin, caretakers, or guardians on the behalf of the minors/children participants was not applicable in our study.

## Results

### Characteristics of the Study Population at Baseline and at Endpoint

The socio-demographic characteristics and medical data of the study population at baseline and endpoint are shown in table 1. Briefly, the median age of the participants was 35 years [IQR: 29–44]; 70.2% (99/141) of the population was female, and ∼75% of the overall study population were on an advanced stage of disease (median CD4: 125 cells/mm^3^ [IQR: 51–208]) at enrollment, indicating a delay in treatment initiation. Up to 97% patients (137/141) received a standard national first-line ART regimen, the others being treated with protease inhibitor (lopinavir/ritonavir) containing regimens due to resistance to non-nucleoside reverse transcriptase inhibitors.

**Table 1:** Socio-demographic and medical data of the study population.

Patients	M0 (141)	M12 (76)
Sex (Male/Female)	42/99	21/55
Median CD4 (IQR)	125 (51–208)	Not applicable
Exposure to ARV for PMTCT	2	Not applicable
Still on ART	Not applicable	76
3TC+AZT+NVP/EFV	129	44
3TC+d4T+NVP/EFV	9	31
Other HAART	3 (2 on protease inhibitors)	1
Treatment interruption	Not applicable	0
Deceased	Not applicable	13
Lost-to-follow-up (LTFU)	Not applicable	47
Transferred out	Not applicable	5
>90% Adherence to ART in the past 30 days	Not applicable	93,4%
Treatment switch within first-line ART	Not applicable	29 (20 due to drug shortages)
Change to second line ART	Not applicable	0

**Legend** **table 1:** ART: Antiretroviral therapy; HAART: Highly Active Antiretroviral therapy;

PMTCT: prevention of mother-to-child transmission; IQR: Interquartile range.

At endpoint (M_12_), 72% (55/76) of participants were female. Despite the fact that 44.7% (34/76: indicating patient’s delay in responding to routine medical appointment) of the endpoint attendees showed-up during the additional period of three-months dedicated to monitor cases of LFTU, the majority (93.4%; 71/76) reported a good level of adherence to treatment (i.e. self-reported adherence), and 38% (29/76) had experienced a treatment switch within first line ART (mainly due to drug shortages), and no case of treatment change from first- to second-line drug regimens was recorded; as reported in table 1.

### Prevalence of HIV Drug Resistance at Baseline (M_0_)

At M_0_, 139/141 protease-reverse transcriptase samples were successfully genotyped, giving a sequencing performance of ∼99%. Sequences from 5 patients were identified with drug resistance-associated mutations, giving a prevalence of 3.6% (5/139) HIVDR, among which 3 resistance mutations to protease inhibitors (M46I, G73A, L90LM); 2 to nucleoside reverse transcriptase inhibitors (M184V), T215F); and 3 to non-nucleoside reverse transcriptase inhibitors (K103N, Y181C, Y181YC, M230ML). These 5 patients were enrolled on first-line ART; at endpoint, 2 of them were LTFU while the 3 others showed good virologic response; as shown in table 2.

**Table 2:** HIV drug resistance at enrollment on ART.

Sample ID	PI	NRTI	NNRTI	HAART	VL (M12)	Overall HIVDR
MR046	M46I	–	–	NVP-3TC-AZT	143 copies/ml	
MR067	–	M184V, T215F	Y181C	NVP-3TC-AZT	LTFU	
MR078	–	–	K103N, Y181C	NVP-3TC-d4T	LTFU	
MR100	G73A, L90LM	–	–	NVP-3TC-AZT	VL not detected	
MR105	–	–	M230ML	EFV-3TC-AZT	VL not detected	
TOTAL NUMBER OF PATIENTS ANALYZED: 139						3.6% (<5%)

**Legend** **table 2**: PI: protease inhibitor; NRTI: nucleoside reverse transcriptase inhibitor; NNRTI: non-nucleoside reverse transcriptase inhibitor; HAART: highly active antiretroviral therapy; VL: viral load; LTFU: lost to follow-up; NVP: nevirapine; 3TC: lamivudine; AZT: zidovudine; d4T: stavudine; EFV: efavirenz.

### Subtypes of HIV-1 Protease-reverse Transcriptase Sequences

Samples were from patients infected with HIV-1 group M; with CRF02_AG being the most prevailing clade (71.2%; 99/139), followed by CRF22_01A1 (11.5%; 16/139), then subtype D (5%; 7/139), CRF11-cpx (3.6; 5/139), CRF36 (3.6; 5/139), sub-subtype F2 (2.1%; 3/139), subtype G (2.1%; 3/139), and unclassified recombinant.

### Virologic Response and HIV Drug Resistance at Endpoint (M_12_)

At endpoint, 13 and 5 patients were reported respectively as dead and transferred out of the HIV clinic; giving an expected number of 123 living attendees after 12 months of ART. Surprisingly, up to 47 out of the expected 123 failed to show-up for the endpoint appointment, giving a high rate of patient LTFU (38.20%; 47/123) after one-year of ART initiation. Therefore, the rate of patient retention on first-line ART after one-year was poor (62.80%, 76/123), as compared to the WHO-required threshold of 70%.

Out of the 76 attendees on ART at M_12_, 69.73% (53/76) had an undetectable viremia (plasma HIV-RNA <40 copies/ml); 90.80% (69/76) experienced virologic success (plasma HIV-RNA <1000 copies/ml), an outcome above the WHO-required threshold of ≥70% (See Table 3 for viral load distribution per category). A total of 7 patients experienced virologic failure (plasma HIV-RNA ≥1000 copies/ml), among which 3 of them with plasma HIV-RNA of 1,188; 151,418; and 453,666 copies/ml were surprisingly without any DRMs (probably due to non-reported poor adherence or ART interruption, since adherence was solely self reported); whereas the 4 others harbored viruses with DRMs, giving an overall HIVDR prevalence of 5.3% (4/76) at M_12_. Specifically, the most frequent mutations were M184V/I (4/4) conferring high level resistance to 2 NRTIs (lamivudine and emtricitabine), K103K/N (3/4) conferring high level resistance 2 NNRTIs (nevirapine and efavirenz), while only one mutation to thymidine analogs (mainly zidovudine and stavudine) was identified. Resistance-associated mutations to protease inhibitors (backbones for second line ART regimens) were not detected at M_12_ (see Table 4).

**Table 3:** Virologic outcome at the study endpoint (M12).

Viral load classification	Number of patients	Median value [IQR]	Range (min-max)
Not detectable	31	NA	NA
<40 copies/ml	22	NA	NA
≥40– <1000 copies/ml	16	113 [65–238]	40–751
≥1000 copies/ml	7	68624 [6919–453666]	1188–525142
Total number of patients	76		

Legend: NA: not applicable.

**Table 4:** Drug resistance-associated mutations at 12 months of ART.

Sample ID	PI	NRTI	NNRTI	HIVDR prevalence
MR2003	–	M184V	G190A+H221HY	
MR2040	–	M184V	K103N	
MR2086	–	M184I	K103N+Y188H	
MR2136	–	M184V+T215ST	K101EK+K103KN	
TOTAL NUMBER OF PATIENTS ANALYZED: 76				5.3% (≥5%)

**Legend** **table 4**: PI: protease inhibitor; NRTI: nucleoside reverse transcriptase inhibitor; NNRTI: non-nucleoside reverse transcriptase inhibitor; HIVDR: HIV Drug Resistance.

Though our study strength is hampered by the high rate of LTFU at endpoint, baseline and endpoint data showed a non-significant increase in HIVDR emergence (3.6% to 5.3%; p = 0.8196), while protease inhibitor resistance-associated mutations identified at M_0_ were suppressed at M_12_ (see table 2), thus supporting a possible effectiveness of national first- and second- line regimens, concordant with WHO standardized ART recommendations for LRS.

## Discussion

The increasing risk of emerging drug resistant viruses in the developing world suggests HIVDR monitoring as an important component to support patient adherence and programmatic performances, as such data may be useful for patient management in assessing the effectiveness of 1^st^- and 2^nd^-line regimens [11]. Some site-specific factors affecting ARV treatment outcomes were addressed in the present study.

To efficiently help in preventing HIVDR emergence, our investigation would firstly recommend earlier ART initiation and removal of barriers to patient retention on care. Of note, our baseline data revealed that about 75% of patients were already at a severely immune-compromised status (CD4≤200 cells/mm^3^); thereby indicating a potential delay of ART initiation within the Cameroonian context [25]. Such delays are generally due to late HIV diagnosis at the advent of a disease that motivated a medical consultation. Expanding HIV screening (sensitization campaigns) and timely monitoring of untreated patients may reduce this gap; as only half of ART-eligible patients were treated in the country [17], [26]. Since deaths due to AIDS-related conditions (i.e. advanced stage of disease) are associated with delayed referral of patients, the observed delay in ART initiation may have also contributed to the observed mortality (9.2%) and LTFU (≈40%) [25]. Despite this high LTFU, patients on care after M_12_ were high adherent to ART, which in turns support the low rate of HIVDR observed at endpoint. Nonetheless, the high viral load (plasma HIV RNA >1000 copies/ml) shown by some patients harboring wild viruses makes questionable the efficiency of self-reported adherence. Pill count or medication possession ratio may serve as suitable alternatives to evaluate adherence in RLS. In contrast to our finding, a previous study showed a low rate (≈10%) of patients with good adherence in another Cameroonian ART clinic [26], confirming that adherence is also site-specific and affected by several variables (quality of service delivery, workload, patient selection, etc) [19], [20]. The higher representativeness of women in our study population simply highlights the feminization of HIV/AIDS epidemics, and supports gender consideration in the pandemic control strategies [17].

The levels of HIVDR remain low before and after one-year of ART initiation, indicating a possible high viral susceptibility to commonly available first- and second-line regimens used for the management of AIDS in Cameroon: these are in adequacy with WHO-standardized and simplified ART regimens for LRS [23]. However, the high rate of LTFU is detrimental when attempting to accurately appreciate the real burden of HIVDR at the population level; as a patient LTFU is known to possibly harbor HIVDR [23]. Practically, LTFU and delays in clinic appointments could be favored by the costs for medical consultation (at the patient’s responsibility) and laboratory tests (partly subsidized) [26]. More so, treatment switches within first line ART were mainly due to drug shortages (69%; 20/29), a programmatic factor known to be associated with risks of HIVDR emergence, and previously reported nationwide [19], [20]. In addition, heavy workload (physician/patients ratio: 1/180) and poor involvement of community relay agents were also indicated as risk factors of HIVDR [19], [20]. The workload in our study reflects the current disparity between LRS (2–4 workers/1,000 patients) and rich countries (18–23 workers/1,000 patients) [27], encouraging task shifting and community empowerment [21], [28]; alongside subsidizing costs for routine monitoring [19], [28].

The non-significant increment of HIVDR (1.7%) after one-year is concordant to previous reports in Cameroon and in the entire west- and central-Africa regions (3%); contrast to the southern (14%) and eastern (29%) Africa regions [11–13, 18 and 22]. In spite the possibility of a transmitted PI drug resistant virus, these PI-mutations observed at baseline could not preclude previous exposure ART. Of note, detected DRMs at M_12_ were without major effect on second line drug regimens used nationwide, indicating a potential effectiveness of current national HIV/AIDS treatment guidelines. HIVDR surveillance and monitoring is therefore useful informative tool for ART policy-making in LRS [9], [10], [23]. Further studies are needed to ascertain the significance of LTFU on the flourish of HIVDR [19], [20], [26]. More so, the growing risk of HIVDR requires a wider availability of CD4 cells count for a timely ART initiation (i.e. using point-of-care assays), a rapid implementation of point-of-care HIV-1 viral load and affordable GRT assays in LRS. Interestingly, the high failure rate of commercial HIV-1 genotyping kits in sub-Saharan Africa suggests the need of cost-effective in-house sequencing protocols [14], [15], [23].

With the goal to eliminate new HIV infections (especially among infants and children) by 2015 [3] and to ensure the long-term effectiveness of ART in children, pediatric HIVDR may also become another important component of the national HIVDR strategic plan in LRS [12]. The current WHO-HIVDR monitoring and surveillance package should also evaluate the burden of HIVDR among heavily or long-term ART-experienced patients in LRS [21], in order to identify inappropriate treatment switches, to identify time-to-treatment failure and the most effective second- and third-line ART options in LRS [28].

For the Cameroonian ART program, the surveillance of HIVDR remains a national health priority, the national AIDS control program should implement approaches to ensure on-time ART initiation, to limit the rate of LTFU, to ensure continuity in drug supply, to improve adherence, and to further subsidize management cost. Despite the high rate of possible HIVDR (i.e. LTFU), current first- and second-line ART regimens should be continued, while surveillance of acquired HIVDR routinely ascertained.

### Study Limitations

Given the uncertainty in terms of outcomes among those patients LTFU and the likelihood that this group harbors a larger proportion of detectable VL and a possible high rate of HIVDR, the recommendations generated from our findings have limited strengths for policy-making. For instance, if only 5 of the 47 LTFU (10.6%) subjects harbored HIVDR, the actual prevalence at the follow-up time-point would be the double of the baseline prevalence [i.e. (4+5)/123 = 7.3% versus 3.6% at baseline], leading eventually to a different conclusion about ART program performance. This setback (LTFU) in monitoring cohort of patients may require a revision of the WHO HIVDR monitoring strategy in RLS. Henceforth, designing cross-sectional studies for the surveillance of acquired HIVDR at M_12_, M_24_, M_36_, M_48_, would be more achievable.

## Conclusion

This ever first longitudinal HIVDR monitoring survey in Cameroon reports low levels of HIVDR, suggesting a possible effectiveness of current standard ART regimens. However the possible high rate of HIVDR among LTFUs limited the strengths of our findings; this latter suggests the need of operational research among LTFUs to ascertain their real contribution on HIVDR emergence.

Strategies toward an improved adherence, reduced workload by task shifting, continuous drug supply, community empowerment, and subsidized costs for routine patient management, are urgently needed. Finally, revising the WHO-approach for HIVDR monitoring in RLS may help in the achievement of greater evidence-based ART policies.
